# Prediction of heart failure patients with distinct left ventricular ejection fraction levels using circadian ECG features and machine learning

**DOI:** 10.1371/journal.pone.0302639

**Published:** 2024-05-13

**Authors:** Sona M. Al Younis, Leontios J. Hadjileontiadis, Ahsan H. Khandoker, Cesare Stefanini, Stergios Soulaidopoulos, Petros Arsenos, Ioannis Doundoulakis, Konstantinos A. Gatzoulis, Konstantinos Tsioufis

**Affiliations:** 1 Department of Biomedical Engineering, Healthcare Engineering Innovation Centre (HEIC), Khalifa University, Abu Dhabi, United Arab Emirates; 2 Department of Electrical and Computer Engineering, Aristotle University of Thessaloniki, Thessaloniki, Greece; 3 Creative Engineering Design Lab at the BioRobotics Institute, Applied Experimental Sciences Scuola Superiore Sant’Anna, Pontedera (Pisa), Italy; 4 First Cardiology Department, School of Medicine, “Hippokration” General Hospital, National and Kapodistrian University of Athens, Athens, Greece; The Open University, UNITED KINGDOM

## Abstract

Heart failure (HF) encompasses a diverse clinical spectrum, including instances of transient HF or HF with recovered ejection fraction, alongside persistent cases. This dynamic condition exhibits a growing prevalence and entails substantial healthcare expenditures, with anticipated escalation in the future. It is essential to classify HF patients into three groups based on their ejection fraction: reduced (HFrEF), mid-range (HFmEF), and preserved (HFpEF), such as for diagnosis, risk assessment, treatment choice, and the ongoing monitoring of heart failure. Nevertheless, obtaining a definitive prediction poses challenges, requiring the reliance on echocardiography. On the contrary, an electrocardiogram (ECG) provides a straightforward, quick, continuous assessment of the patient’s cardiac rhythm, serving as a cost-effective adjunct to echocardiography. In this research, we evaluate several machine learning (ML)-based classification models, such as K-nearest neighbors (KNN), neural networks (NN), support vector machines (SVM), and decision trees (TREE), to classify left ventricular ejection fraction (LVEF) for three categories of HF patients at hourly intervals, using 24-hour ECG recordings. Information from heterogeneous group of 303 heart failure patients, encompassing HFpEF, HFmEF, or HFrEF classes, was acquired from a multicenter dataset involving both American and Greek populations. Features extracted from ECG data were employed to train the aforementioned ML classification models, with the training occurring in one-hour intervals. To optimize the classification of LVEF levels in coronary artery disease (CAD) patients, a nested cross-validation approach was employed for hyperparameter tuning. HF patients were best classified using TREE and KNN models, with an overall accuracy of 91.2% and 90.9%, and average area under the curve of the receiver operating characteristics (AUROC) of 0.98, and 0.99, respectively. Furthermore, according to the experimental findings, the time periods of midnight-1 am, 8–9 am, and 10–11 pm were the ones that contributed to the highest classification accuracy. The results pave the way for creating an automated screening system tailored for patients with CAD, utilizing optimal measurement timings aligned with their circadian cycles.

## Introduction

Heart failure (HF) is a life-threatening disease that impacts approximately 63 million individuals on a global scale [1]. It represents a growing challenge for cardiologists, as they encounter an annual influx of 3.5 million new patients [2]. At the age of 55, the chances of experiencing heart failure during one’s lifetime are 29% for men and 33% for women, respectively [3]. Based on estimations, about 480,000 adults aged 18 and above are afflicted by heart failure, making up 2.1% of the adult population [4]. It has been conventionally characterized as reduced heart efficacy in pumping and/or receiving blood. Conversely, it may be defined as an irregularity in the heart’s function or structure, leading to an inadequate cardiac output. In certain situations, an adequate cardiac output can be resulted through compensatory neurohormonal mechanisms and an increase in left ventricular filling pressure. Left Ventricle Ejection Fraction (LVEF) levels, is a hemodynamic term for the fraction of ventricular volume ejected per heartbeat [5]. Furthermore, LVEF has been emphasized as a significant marker for diagnosing, predicting outcomes, and managing patients with heart failure [6, 7], that encompasses physical examinations, patient history reviews, and clinical tests [8].

Although there are some variations in the heart failure definitions currently found in the practice guidelines of different organizations (e.g., American Society of Echocardiography and the European Association of Cardiovascular Imaging (ASE/EACVI) [9, 10], American College of Cardiology (ACC)/American Heart Association (AHA) [11], Heart Failure Association (HFA)/European Society of Cardiology (ESC) [12], and the Japanese Heart Failure Society (JHFS) [13]), the fundamental concepts and criteria for classifying heart failure into distinct stages are rooted in the assessment of the condition’s severity, progression, and the observable signs and symptoms. These symptoms encompass issues, such as edema/fluid retention, dyspnea, reduced tolerance for physical activity, and fatigue. Furthermore, these guidelines stress the importance of having structural or functional heart disease as a prerequisite for making a diagnosis. Coronary artery disease (CAD) stands as the primary contributor to heart failure, with hypertension, diabetes, valvular heart disease, and cardiomyopathy following as significant underlying causes [14].

As per the guidelines provided by the ASE/EACVI [9, 10], heart failure with a decreased ejection fraction, or systolic dysfunction (HFrEF), is the clinical presentation of symptoms associated with heart failure, characterized by a resulting LVEF below 50%. A diastolic dysfunction, usually termed heart failure with preserved ejection fraction (HFpEF), is identified when the measured Ejection Fraction (EF) exceeds 55%. However, in cases where the LVEF shows a slight reduction (falling within the range of 50–55%), it is known as heart failure with mid-range ejection fraction (HFmEF). Due to the HF aetiology, the HFmEF category’s narrower range is seen as a changeable criterion for this category. Alternative standards, including those set by the ESC [12] and JHFS [13], recommend distinct threshold values for heart failure classification. In these guidelines, the cut-off for HFrEF can be as low as 40%. There are no strict rules, and the treatment is only tangentially related to LVEF levels and clinical presentation, according to the literature that is currently available. Nevertheless, based on the ESC criteria, patients in the mid-range group with an LVEF of 40–49% showed that 90% of patients either improved or became worse, with only 10% of cases remaining unaltered. LVEF is evaluated from computerized tomography (CT) [15], cardiac catheterization [16], and nuclear medicine scans [17]. Nevertheless, cardiovascular magnetic resonance (CMR) [18] imaging and echocardiography [19] stand out as the most dependable and extensively utilized techniques for assessing LVEF. While these techniques offer high estimation accuracy and utilize non-ionizing radiations, they come with certain limitations. These include factors, i.e., accessibility and cost, patient contraindications, dependence on operator skills, patient cooperation, challenges in interpreting the obtained images, and time requirements.

Electrocardiography (ECG) serves as a valuable adjunct, offering accessibility and cost-effectiveness in the assessment of LVEF. The utilization of ECG for LVEF evaluation is justified by its proficiency in capturing essential physiological correlations indicative of cardiac performance. ECG characteristics, including QTc interval, QRS duration, abnormalities in T-wave patterns, and alterations in the ST segment, serve as indicators of irregularities in electrical conduction, anomalies in ventricular repolarization, and myocardial ischemia. These electrical manifestations are intricately connected to the mechanical processes that regulate the contraction and relaxation of the ventricles. Significantly, changes in LVEF can impact cardiac electrical activities, leading to variations in ECG patterns. Consequently, through a thorough analysis of ECG signals and their derived parameters, it becomes feasible to understand the intricate relationship between these electrical indicators and LVEF. This approach provides valuable insights into cardiac function without the need for more invasive or costly procedures.

Numerous cardiovascular conditions, such as atrial fibrillation, myocardial infarction, congestive heart failure, and premature contractions in the atria or ventricles can be identified through the analysis of ECG data [20–25]. As a result, the automatic and precise analysis of ECG data has emerged as a prominent area of study, particularly with the utilization of artificial intelligence (AI) tools. Automatic ECG analysis has utilized established diagnostic principles as guidelines. This procedure encompasses two stages in which human specialists extract meaningful features from raw electrocardiogram (ECG) data. These features can be categorized into various classes, including statistical parameters (such as heart rate variability), density histograms and coefficients of variation, time-domain features, sample entropy, and frequency-domain features. Leveraging ECG data and machine learning (ML) techniques, many researchers have successfully differentiated patients with congestive HF from those with normal cardiac conditions [26, 27]. Conversely, deep learning, utilizing a single lead ECG signal, has been employed to classify HF patients into categories such as congestive HF, Coronary Artery Disease (CAD), and Myocardial Infarction (MI) patients [20].

Nevertheless, the intricate connection between circadian ECG wave characteristics and the estimation of LVEF in HF patients is not yet fully understood. Additionally, providing a more accessible approach for aiding in the diagnoses of HF patients with HFpEF, HFmEF, and HFpEF that does not require extensive expertise or costly equipment would be highly beneficial. In a similar vein, the utilization of ML techniques, including deep learning, can be pivotal in unravelling the complexities of ECG features within patient records, ultimately enhancing the assessment of HF. Therefore, the aims of this study are:

1. To investigate the capability of ML classification models trained on features derived from the ECG waveform.
2. Consistent with the ASE/EACVI guidelines, to precisely categorize heart failure patients into HFpEF, HfmEF, and HfrEF classifications.
3. To examine the cardiovascular system dynamics in heart failure patients on an hourly basis throughout a 24-hour day.
4. To highlight the circadian functionality of the heart over a 24-hour period (correlated with ECG features) with the aim of proposing optimal times for accurate heart failure classification. This provides a viable screening strategy with the most favourable opportunities for intervening and preventing the progression of heart failure.

## Materials and methods

### Dataset and patients’ enrollment

**Fig 1:**
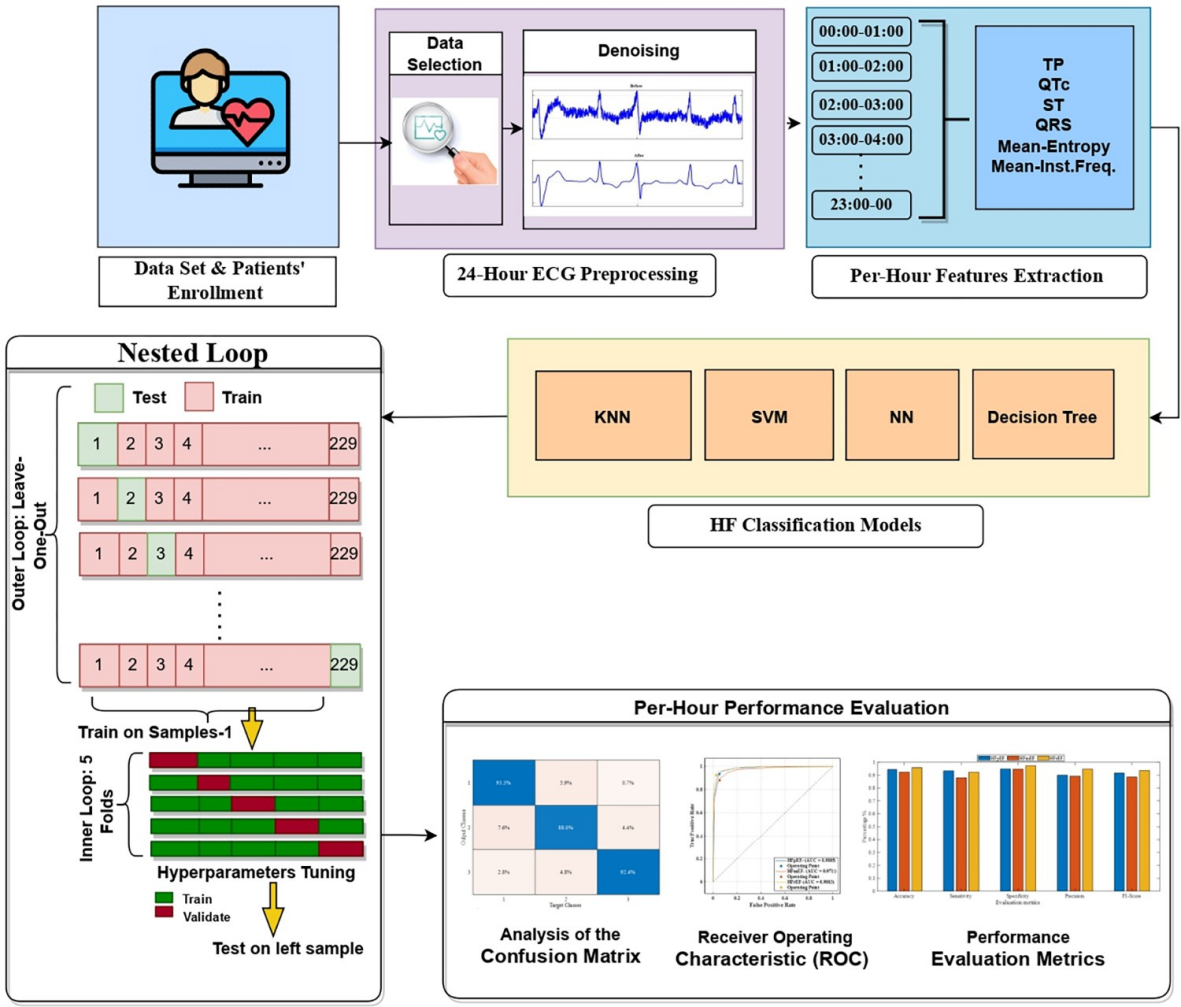
A visual representation of the entire process carried out in this study.

The comprehensive methodology employed in this study is depicted in Fig 1. The research utilized two datasets containing clinical information from cohorts of American and Greek patients. Encompassing individuals with heart failure, specifically CAD, and spanning an age range from 33 to 88 years (n = 303), both datasets were incorporated. Following the ASE/EACVI guidelines, the patients were categorized into 92 with HFmEF, 129 with HFpEF, and 82 with heart failure with HfrEF. Choosing ASE/EACVI guidelines enhances the comparability of our study’s results with those of other research endeavors that have followed similar criteria [50–64]. This comparability is crucial for validating our findings and contributing to the cumulative knowledge within the scientific community. Moreover, focusing on the narrower range of classifying mid-range HF patients is valuable for informing targeted interventions and treatment strategies, and ASE/EACVI guidelines offer a comprehensive framework for such classifications.

The American patient cohort for this study was identified through the utilization in the University of Rochester Medical Centre’s Telemetric and Holter ECG Warehouse (THEW), of the Intercity Digital Electrocardiography (ECG) Alliance (IDEAL) study archives [28]. The enrolment protocol for the database adhered to the principles of the Declaration of Helsinki and Title 45, U.S. Code of Federal Regulations, Part 46, Protection of Human Subjects (revised: November 13, 2001, effective: December 13, 2001). Moreover, the IDEAL protocol received approval at the University of Rochester from the research subject review board [29]. Prior to participation in the trial, each patient provided written consent.

To be eligible for enrolment in the IDEAL study, participants had to satisfy the following criteria: (a) presenting evidence of either a previous myocardial infarction (MI) or ischemia brought on by exercise; (b) having ischemic heart disease in a steady stage for a minimum of two months after the last event; (c) not having received a diagnosis of congenital heart failure; and (d) maintaining sinus rhythm. In addition, individuals with enlarged heart muscle (left ventricular diameter (LVD) exceeding 60 mm and an ejection fraction below 40%), history of coronary artery bypass grafting (CABG) surgery, as well as those with cerebral, severe hepatic, or malignant problems, were deemed ineligible for the trial. All participants underwent an echocardiography examination at the time of the enrollment to ascertain their LVEF levels. There was an initial resting supine period for a 20 minutes’ duration before starting the ambulatory recordinga 24-hour ECG test that was conducted on each participant using three pseudo-orthogonal lead configurations (X, Y, and Z) that correspond to limb lead I, augmented limb lead aVF, and precordial lead V3, respectively.

Individuals participating in the Greek patient cohort were sourced from seven cardiology departments as part of the PRESERVE EF trial in Greece [30]. The patient enrolment protocol for the PRESERVE EF study (clinicaltrials.gov identifier NCT02124018) obtained clearance from the Hellenic Society of Cardiology’s ethics committee at each of Greece’s seven accredited cardiology departments. The Hellenic Society of Cardiology created and managed a database [31]. Before enrolling in the trial at each cardiology department, every patient provided signed consent. To be eligible for enrolment, patients were required to meet the following criteria: (a) having a MI confirmed post-angiographically 90 days after any coronary artery bypass graft (CABG) surgeries, or 40 days after the event of primary percutaneous coronary intervention (PCI) if applicable; (b) undergoing revascularization; (c) not undergoing revascularization but lacking remarks of any ischemia in the preceding six months; and (d) following optimal and tolerated medical therapy in the case of preceding revascularization (PCI or CABG).

Furthermore, any patient with a secondary prevention indication for implantable cardioverter defibrillator (ICD) implantation, permanent pacemaker, persistent, long-standing persistent, and permanent atrial fibrillation, any neurological symptoms of syncope or pre-syncope within the last 6 months, and presence of any systemic illnesses such as liver failure, renal diseases, rheumatic diseases, thyroid dysfunction, and cancer was excluded from the study. All patients underwent echocardiographic tests following the recommendations of the American Society of Echocardiography, and then a GE Healthcare GETEMED CardioDay Holter system (including CardioMem CM4000 recorder and CardioDay v. 2.4 software, GE Healthcare, Fairfield, CT, USA) was utilized, followed by a 45-min high-resolution digital ECG recording.

All patients were in stable condition prior to their enrollment time. The acquired 24-hour ECG Holter recordings from illegible individuals were sampled at 200 Hz. Patients with missing recordings of an hour or more were not included in the study. Consequently, the dataset comprised only 229 patients, categorized into 105 HFpEF, 60 HFmEF, and 64 HFrEF following the ASE/EACVI guidelines. Additional details about the chosen dataset can be found in Table 1. The Kruskal-Wallis test, a non-parametric method, was employed to assess statistical differences among the three groups for continuous variables. For categorical variables, a chi-squared (χ^2^) test was conducted to investigate dependencies on individual Left Ventricular Ejection Fraction (LVEF) categories. A significant difference (p < 0.05) indicates statistically significant of discriminating between the three LVEF categories.

**Table 1:** Clinical attributes of the patients with HF categorized according to LVEF classifications.

Clinical Variables	Overall Subjects	LVEF Classes			p-value
		HFpEF (n =)	HFmEF (n =)	HFrEF (n =)	
**Patients (n)**	229	105	60	64	-
**LVEF (%)**	25–82	56–82	50–55	25–49	<0.001
**(Mean±Std)**	(56.16±11.79)	(66.63±6.7)	(52.82±2.24)	(42.12±5.52)	-
**Gender (M/F)**	196/33	90/15	49/11	57/7	0.237
**Age (yrs.)**	35–88	35–79	40–88	40–80	0.110
**(Mean±Std)**	(58.29±10.75)	(57.14±10.4)	(60.75±10.88)	(57.86±10.97)	-
BMI (kg/m^2^)	17.99–37.89	19.72–36.33	17.99–37.65	20.8–37.89	0.133
**(Mean±Std)**	(27.15±3.62)	(26.66±3.44)	(27.49±3.89)	(27.61±3.59)	-
**Smoking (Yes/No)**	167/62	72/33	48/12	47/17	0.757
**Diabetes (Yes/No)**	29/200	7/98	7/53	15/49	<0.001
**Hypertension (Yes/No)**	113/116	51/54	31/29	31/33	0.587
**Angina (Yes/No)**	159/70	83/22	37/23	39/25	0.189
**VT (Yes/No)**	19/210	9/96	2/58	8/56	0.088
**Prior-MI (Yes/No)**	150/79	54/51	44/16	52/12	<0.001
**Medications (Anti-Arrhythmic, Beta-Blockers, ACE Inhibitor, Diuretic)**					
**Beta-Blockers (Yes/No)**	179/50	80/25	46/14	53/11	0.478
**ACE Inhibitor (Yes/No)**	68/161	30/75	15/45	23/41	0.611
**Anti-Arrhythmic (Yes/No)**	7/222	3/102	4/56	0/64	0.166
**Diuretic (Yes/No)**	42/187	1/104	19/41	22/42	<0.001

BMI: Body Mass Index, VT: Ventricle Tachycardia, MI: Myocardial Infarction, ACE: Angiotensin converting enzyme.

### Pre-processing and extracting features

The database includes an annotation file with the R-peak locations for each recording, and the RR interval data were manually reviewed to remove ectopic beats, resulting in biosignals of normal beats (NN intervals). To determine the running TP, QT, ST interval, and QRS width within a measurement window, we considered a sufficient number of heartbeats. The annotated NN interval data underwent a Single Cosinor Analysis fitting algorithm [32] to ensure a 24-hour circadian cycle for all recordings. The reference achrophase angle was set at 0°, representing the 00:00 hour. Each hour corresponds to a 15° phase angle increase from the reference angle. After obtaining the starting hour, the 24-hour data was standardized to commence at midnight for all patients. The SDROM-ADF filter was further applied to eliminate noise from each hour of the Holter ECG data [32]. We took the average of the relevant intervals through this hourly window to capture the variations over the entire 24-hour period accurately. Frequency and time-domain characteristics were derived from the ECGs after the de-noising process, as elaborated below. Complex methodologies have been employed to precisely analyze the P-QRS-T components of the ECG waveform. A standard ECG consists of P wave, QRS complex, and T wave. The QRS complex is generated by the electric currents generated during the depolarization of the ventricles prior to contraction, as well as the spread of depolarization throughout the ventricular myocardium. Conversely, the P wave is formed by the electrical currents produced as the depolarization of atria before contracting. In clinical analysis, changes in the ST-T segment are crucial indicators of various cardiac conditions. ST-segment elevation or depression, for example, can signal myocardial infarction or ischemia. T-wave abnormalities may be associated with electrolyte imbalances, myocardial ischemia, or other cardiac issues. Detailed definitions and descriptions of the ECG features are presented in Table 2.

**Table 2:** Descriptions of ECG characteristics.

ECG Feature	Definition
**TP (ms)**	Denotes the period when the ventricles are repolarized and at rest. Variations in the TP interval may show abnormalities in repolarization, which can be linked to impaired cardiac function in HF patients.
**QT (ms)**	Reflects the total time for ventricular electrical activity, including both depolarization and repolarization. It is influenced by heart rate and can be used to assess the risk of arrhythmias.
**ST-T (ms)**	ST represents the early part of ventricular repolarization. The T wave follows the ST segment and represents the later phase of ventricular repolarization.
**QRS (ms)**	Represents the contraction of the ventricles, responsible for pumping blood to the pulmonary and systemic circulation.
**Mean-Entropy**	Used to analyse the complexity and unpredictability of the ECG. Higher mean entropy might indicate more irregular and complex patterns, which could be linked to various conditions or changes in the cardiac function.
**Mean-Instant Frequency**	A measure of how rapidly the frequency of a signal is changing at a particular point in time. Changes in instant frequency could relate to heart rate variability or arrhythmias.

The QT interval exhibits substantial variation based on factors such as age, gender, heart rate, and medication usage. As heart rate decreases, the QT interval tends to lengthen, and as heart rate increases, the QT interval tends to shorten. Correcting the QT interval aims to get a more accurate assessment of the duration of ventricular repolarization, independent of heart rate. Consequently, Bazett’s method is widely employed for its simplicity and significance [33]. RR denotes the R-R interval, and QTc (QTc = QT/sqrt(RR)) represents the corrected QT interval. The Slope Intersect (SI) approach was applied in the automated detection of the QT interval in this study [34]. In Matlab, the Pan-Tompkins ECG QRS detector was utilized to identify the QRS complex [35]. Additionally, the ECG Matlab Toolbox [36] was employed to extract TP, PR, and ST-T. The initial moment of the power spectrogram, calculated over 255-time frames, was employed to estimate the instantaneous frequency of the signal. Furthermore, the spectral entropy of the power spectrogram was computed to assess the flatness and spikiness of the signal’s spectrum.

### Machine learning methodologies and training configurations

For an efficient classification process, the capability to train and classify patients into HFpEF, HFmEF, and HFrEF categories using ML algorithms is essential. In this research, four models—Support Vector Machine (SVM), K-Nearest Neighbors (KNN), Neural Network (NN), and Decision Tree (TREE)—were employed to evaluate the effectiveness of diverse machine learning approaches. Accuracy was the primary metric optimized for our models, this choice would align with the nature of the LVEF prediction task and the goals of the research. In our study, the hyperparameter values were selected based on a combination of domain expertise and experimentation. Specifically, we aimed to cover a range of values that are commonly recommended for the respective algorithms used in our models. These values were chosen to ensure comprehensive coverage of the parameter space while also considering computational feasibility.

#### 1)SVM

Typically, a standardized and flexible machine learning approach is employed for addressing classification problems. Support vectors delineate the margins of hyperplanes, and their identification involves an optimization process incorporating a regulated objective function by a constraint and error term, utilizing Lagrangian relaxation [37]. The intricacy of the SVM classification task is contingent on the count of SV rather than the input space dimensionality. The support vector numbers retained from the initial dataset is contingent on the data and fluctuates according to data complexity, encompassing factors such as data dimensionality and class reparability [38].

SVMs are powerful models with several hyperparameters that significantly influence their performance [39]. The primary hyperparameters to adjust are the kernel type and the regularization parameter;

1. Kernel type: The kernel determines the type of decision boundary the SVM will use. common choices include radial basis function (RBF), polynomial, linear, and sigmoid kernels.
2. Regularization parameter (C): C is the trade-off parameter to control regularization between achieving a boundary with smooth decision and correct classification of the training points. Higher values of C lead in a more complex model that may lead to overfitting, while lower values may lead to under fitting.

In this study, the kernel types are selected to be linear, polynomial, RBF, and sigmoid. The C values are defined in the range of: [0.1, 10, 100]. We use the default settings in Matlab for the rest of the other hyperparameters.

#### 2) NN

The intrinsic capacity of neural networks to acquire sophisticated representations and capture intricate relationships renders them highly effective tools for classification assignments. Their versatility, non-linear characteristics, and capability for representation learning are pivotal factors contributing to their triumph in diverse domains such as natural language processing, image recognition, and healthcare. NN specifically multi-layer perceptron (MLP) architecture are comprised of interconnected nodes organized into layers, with an input layer, an output layer, and one or more hidden layers. Associated with each connection between nodes is a weight, and each node is equipped with an activation function that dictates its output [40]. Activation functions introduce a non-linear element to the model, enabling it to grasp and express non-linear relationships within the data. This becomes essential when dealing with intricate real-world datasets, as linear models may prove inadequate in capturing their complexities [41].

While NN encompass numerous hyperparameters, this study specifically focuses on tuning a select set of hyperparameters, namely:

1. Hidden layers: The layers between the output and input layers, adjusting the number of hidden layers can impact the network’s capacity to learn complex patterns. Values explored: [1, 2, 3].
2. Hidden units: The neurons (number of nodes) within each hidden layer. Modifying the number of hidden units influences the model’s capacity to capture and represent features in the data. Values explored in this study: [10, 50, 100].
3. Activation function: The non-linear function applied to each node’s output in a layer, introducing non-linearity to the model. Different activation functions offer distinct non-linear properties, impacting the network’s ability to learn and generalize. Values explored: [relu, sigmoid].
4. Learning rate: The step size used during the optimization process (e.g., gradient descent) to update the model’s weights. The learning rate determines the magnitude of weight adjustments, influencing the convergence and stability of the training process. Values explored: [0.1, 0.01, 0.001].

By systematically exploring these hyperparameters and their respective values, the study aims to identify the configuration that optimally balances model complexity, learning capacity, and generalization performance. The chosen values within these ranges allow for a comprehensive search over different architectural and optimization configurations, providing insights into the NN’s behaviour on the specific classification task under investigation.

### 3) KNN

K-nearest neighbours is an effective yet simple machine learning algorithm applied for regression and classification tasks. KNN fundamental principle is based on the concept that data points tend to belong to the same class or have similar output values when they exhibit similar properties. It operates on the assumption that proximity in the feature space indicates similarity in the underlying patterns. Each data point is interpreted as a vector in a multi-dimensional feature space [42]. The distance between the new data point and every other point in the dataset is calculated using a distance algorithm such as Euclidean. The ’k’ points with the shortest distances to the new point are identified. In categorization assignments, the algorithm allocates the new point to the class with the highest degree of commonality among its ’k’ closest neighbours [43]. Hyperparameters to Tune in KNN:

1. Number of neighbors (k): The number of nearest neighbours set when making predictions. Choosing ’k’ is crucial; While a bigger ’k’ might make the model more resilient but possibly less sensitive to local patterns, a smaller ’k’ might result in a more flexible model. Values explored: [3, 5, 7, 9]
2. Algorithm: The algorithm used to compute the nearest neighbours. Different algorithms have varying computational complexities and may perform differently based on the dataset size and dimensionality. ’ball_tree’ and ’kd_tree’ are tree-based methods suitable for low-dimensional data, while ’brute’ performs a brute-force search. Values explored: [ball_tree, kd_tree, brute].

#### 4) TREE

For classification tasks, the decision tree algorithm provides a reliable and simple-to-understand machine learning technique. At its core, this algorithm aims to construct a hierarchical set of decision rules by leveraging the features present in the input data. This entails the iterative division of the dataset into subsets, each linked to a distinct decision or result [44]. It is composed of nodes, each representing a decision or a test on a feature. The topmost node is called the root node. It represents the entire dataset. In order to divide the data into subsets, the algorithm chooses the best feature and a related threshold. The selection is based on criteria such as entropy, Gini impurity, or information gain. Subsequent nodes, known as decision nodes or internal nodes, represent the tests applied to the features. Every internal node defines a condition that depends on a threshold and a characteristic.

To make predictions, new data traverses the tree from the root to a leaf node based on the feature values. The class associated with the reached leaf node is assigned as the predicted class. One of the notable advantages of decision trees is their interpretability. The resulting tree structure provides a clear and intuitive representation of the decision-making process. Achieving optimal performance and dependable generalization necessitates meticulous adjustment of hyperparameters and precautions against overfitting. To control the complexity of the tree and reduce the likelihood of overfitting, crucial factors are considered when fine-tuning decision tree hyperparameters. These hyperparameters encompass, among others, maximum depth, minimum samples split, criterion for splitting, minimum samples leaf, and maximum leaf nodes. In the course of hyperparameter tuning, key considerations involve exploring maximum depth values of [5, 10, 20] and selecting between criteria for splitting, namely ’gini’ and ’entropy’ was done in this work.

### Optimized settings

The optimal combination of SVM hyperparameters was identified as C = 10 with a linear kernel type. A moderate C value indicates that the SVM model maintains a low tolerance for errors in the training data, emphasizing a close fit to the training data, even permitting moderate margin violations. Employing a linear kernel, the SVM model endeavors to establish a hyperplane in the feature space that optimally aligns with the data points, simultaneously maximizing the margin between positive and negative support vectors [45]. The simplicity and interpretability of the linear model render it advantageous in situations where the relationship between target variable and input features can be adequately modeled by a linear function.

The found optimal combination for the NN hyperparameters involves a singular hidden layer with 100 hidden units, and the utilized activation function is the Rectified Linear Unit (ReLU), and employing a learning rate of 0.01. A singular hidden layer with an ample number of units (100) allows the NN to capture relationships and intricate patterns within the data. This configuration strikes a balance between model complexity and efficiency, ensuring the network’s capability to learn and represent the features relevant to the classification task. ReLU is widely adopted for its effectiveness to introduce non-linearity to the model, assisting in the identification of intricate connections within the data. Its simplicity and avoidance of the vanishing gradient problem contribute to more effective training, especially in scenarios where intricate feature representations are essential. A moderate learning rate like 0.01 strikes a balance, preventing convergence issues associated with excessively large rates while ensuring efficient convergence [46]. This combination optimally balances model complexity, training efficiency, and the network’s capacity to discern between different heart failure categories, leading to enhanced accuracy in classification.

In the case of KNN, the identified optimal combination involves selecting 5 neighbours (K = 5) and using the k-d tree algorithm for classification. Five neighbours strike a balance between model sensitivity and robustness. A higher value might lead to over smoothing and generalized predictions, while a lower value might result in sensitivity to noise. With K = 5, the model considers a moderate number of neighbours for classification, ensuring a stable yet responsive approach to identifying patterns in the dataset. The k-d tree algorithm efficiently organizes data points in a multidimensional space, enhancing the speed of nearest neighbour search operations [42]. This is particularly beneficial when dealing with a moderate-sized dataset and a specific choice of K (such as 5). The k-d tree helps in quickly identifying the closest neighbours, contributing to faster and more accurate predictions. Finally, the optimal combination for the decision tree hyperparameters, specifically max_depth = 10 and criterion = gini. With max_depth = 10, complex relationships in the data can be observed by the decision tree without becoming overly intricate. This value strikes a balance, allowing the model to generalize well to unseen data while avoiding unnecessary complexity. The criterion parameter determines the measure of impurity used to evaluate the quality of a split. Gini impurity is effective for classification tasks, and with this choice, the decision tree seeks to minimize the Gini impurity at each node [47]. By selecting the split that minimizes impurity, the model ensures that the resulting nodes are more homogenous in terms of class labels, contributing to clearer decision boundaries. Our findings suggest that each classification model demonstrated outstanding performance when optimized with its most effective hyperparameter combination.

### Configuration for training and testing phases

For evaluating the ML models, a nested cross-validation framework (Fig 1) was adopted. In the implemented methodology the outer loop follows the "Leave-One-Out Cross-Validation" (LOOCV) approach, where in each iteration, one patient is withheld for testing, while the remaining (n-1) patients are used for training. This process is then repeated for all n patients in the dataset. LOOCV is advantageous as it maximizes dataset utilization, provides an unbiased evaluation, offers high variability insights, suits small datasets, tests robustness, ensures consistent performance estimation, is applicable to imbalanced datasets, and maintains simplicity in its implementation.

Within the outer loop, there is an inner loop that utilizes a 5-fold cross-validation strategy for hyperparameter tuning. In this inner loop, the (n-1) patients from the training set are further divided into five subsets, and the model is trained and validated across these subsets iteratively. This helps optimize the hyperparameters of the model to enhance its performance.

After the inner loop completes, the model with tuned hyperparameters is tested on the patient left out in the outer loop. This entire process is repeated for each patient in the dataset (n times), and the performance metrics (such as accuracy, precision, recall, etc.) are recorded for each iteration. Finally, the average of these metrics is calculated to provide a comprehensive and representative assessment of the model’s performance across all patients in the dataset. This approach ensures robust evaluation, hyperparameter tuning, and reliable estimation of the model’s generalization capabilities.

Grid search is a specific method within the broader concept of Cartesian product, it introduces practical constraints to make hyperparameter tuning flexible, less computationally expensive, and feasible in real-world scenarios. In our study, grid search technology is included into the nested loop approach to efficiently optimize the model’s hyperparameters. Grid search covers a range of combinations of hyperparameter values by methodically examining a predetermined hyperparameter grid. The model’s performance is assessed based on several hyperparameter combinations at each iteration of the nested loops. This makes it possible to conduct a thorough search for the ideal set of hyperparameters that maximizes the accuracy and generalizability of the model.

Analysis of the prediction confusion matrix, receiver operating characteristic (ROC) curves, and the associated area under the ROC (AUROC) were calculated to assess the performance of the classification models. Additional measures for performance evaluation, such as F1-score, accuracy, precision, sensitivity, and specificity were performed. In our study, we employed a straightforward averaging approach as the model aggregation technique. Specifically, we computed the average performance metrics across all 229 models trained during the LOOCV process. This method enabled us to obtain an aggregated view of the models’ performance across the entire dataset, rather than focusing solely on individual instances.

Furthermore, in the tree classification model, the calculation of feature importance is a crucial aspect of understanding the contribution of the feature to the predictive capability of the model. Feature importance is typically measured by assessing how much each feature reduces the impurity in the model. During the construction of decision trees, features are selected at every node to divide the data based on the chosen criterion (e.g., entropy or Gini impurity). The reduction in impurity achieved by each feature is recorded, and this process is repeated for all nodes in the tree. The more a feature contributes to reducing impurity across the entire tree, the more important it is considered.

In the context of nested cross-validation (e.g., LOOCV and 5-fold cross-validation), feature importance is calculated iteratively over multiple folds. This ensures that the estimation of feature importance remains robust and is not overly influenced by the specific characteristics of a single training-test split. Understanding feature importance provides insights into which features have the most significant impact on the potential of the model to differentiate between different classes (e.g., HFpEF, HFmEF, HFrEF). It aids in feature selection, model interpretation, and potentially highlights key factors influencing the classification accuracy.

## Results

The Intel Core i5-1135G7 (11th Gen) processor with a single GeForce NVIDIA MX350 (2 GB) of RAM was utilized for hourly model validation and training. Because the nested loop strategy was used, each model took 10–20 minutes for the training and testing phases. Table 3 displays the ideal hyperparameter combinations for each model after it has been tuned.

**Table 3:** Classification models and hyperparameters tuning.

Classification Model	Hyperparameters	Best Combination
**SVM**	**Regularization Parameter (C): [0.1, 10, 100]** **Kernel Types: [linear, polynomial, rbf, sigmoid].**	**C Value = 10** **Kernel Type = linear**
**Neural Network**	**Hidden_layers: [1, 2, 3] Hidden_units: [10, 50, 100] Activation function: [sigmoid, relu]** **Learning-rate: [0.1, 0.01, 0.001]**	**Hidden_layers: [1] Hidden_units: [100] Activation function: [relu]** **Learning_rate: [0.01]**
**KNN**	**Number of Neighbours (k): [3, 5, 7, 9]** **Algorithm (algorithm): [ball_tree, kd_tree, brute]**	**K = 5** **Algorithm = kd_tree**
**Decision TREE**	**Maximum Depth (max_depth): [5, 10, 20]** **Criterion for Splitting (criterion):[gini, entropy]**	**max_depth = 10** **criterion = gini**

Table 4 presents a summary of the extracted ECG characteristics (TP, QTc, ST-T, QRS, Mean-Entropy, and Mean-Infrequency) for each of the three groups (HFpEF, HFmEF, and HFrEF) along with statistical comparisons. Regarding the statistical analysis, given the nature of ECG data, which tends to be non-normally distributed, we conducted a test for normality. Specifically, we used the Shapiro-Wilk test to assess the distribution of the data. The results indicated non-normal distributions, justifying the choice of the Kruskal-Wallis test, a non-parametric alternative suitable for such circumstances. The Kruskal-Wallis is used as non-parametric test to assess whether there are statistically significant differences in the ECG features between the three HF classes. Then the post hoc test (pairwise Mann-Whitney U tests) is performed to identify which specific groups differ from each other. The Kruskal-Wallis test and Mann-Whitney U tests are robust to variations in sample sizes and do not assume equal variances. This characteristic makes it suitable for our dataset, which includes different numbers of patients in each HF class (HFpEF: 105, HFmEF: 60, HFrEF: 64).

**Table 4:** ECG features for the three groups (HFpEF, HFmEF, and HFrEF). Statistically significant difference of p-values are indicated in bold.

ECG Feature (±SD)	HFpEF	HFmEF	HFrEF	p-Value			
				HFpEF HFrEF	HFpEF HFmEF	HFmEF HFrEF	HFpEF HFmEF HFrEF
**TP-Interval (ms)**	198±23.5	200±31.3	276±35.2	**0.0021**	**<0.001**	**0.00512**	**0.0014**
**QTc-Interval (ms)**	451±54.1	456±44.2	501±33.7	**<0.001**	**<0.001**	**<0.001**	**<0.001**
**ST-T Interval (ms)**	302±53.6	251±46.7	248±38.9	**<0.001**	**<0.001**	**<0.001**	**<0.001**
**QRS-Interval (ms)**	96±18.3	102±31.1	117±28.5	**<0.001**	**<0.001**	**<0.001**	**<0.001**
**Mean-Entropy**	0.73±0.022	0.83±0.042	0.76±0.036	**0.0081**	**0.0173**	**0.00539**	**0.0015**
**Mean-InsFreq**	38.4±8.7	36.1±10.5	36.3±11.2	**0.0023**	**0.00115**	**0.00343**	**0.0031**

All models were trained using these features. The average accuracy scores for the three HF categories are displayed in Tables 5, 6 and fluctuate throughout a 24-hour period and across various classification models using the optimal combination of hyperparameters. In these tables, each row represents a distinct regression model with the optimal set of hyperparameters, and each column represents a particular point in the 24-hour cycle. In the context of the HFpEF, HFmEF, and HFrEF classification model, accuracy provides a comprehensive evaluation of how well the model performs in correctly categorizing patients into their respective heart failure categories. A higher accuracy value indicates a better overall performance of the model. The tables offer a comprehensive summary of the performance of various classification models throughout the 24-hour period. It facilitates a comparison of different models, highlighting their effectiveness in predicting the HF category at different times of the day.

**Table 5:** Average accuracy values from 00:00–12:00 h, using classification models with best hyperparameters.

Classification Model	Accuracy %											
	00:01	01:02	02:03	03:04	04:05	05:06	06:07	07:08	08:09	09:10	10:11	11:12
**SVM**	**87.8**	87.30	86.60	87.00	87.20	86.00	86.90	87.10	87.00	86.80	87.20	86.50
**Neural Network**	83.80	83.90	83.20	83.80	83.70	82.60	83.30	83.60	**84.30**	83.30	83.70	83.00
**KNN**	90.40	89.70	89.90	89.20	90.20	89.60	88.90	89.20	90.10	89.30	89.40	89.80
**TREE**	90.70	89.90	90.00	89.30	90.10	89.80	88.80	89.40	90.30	89.60	89.40	89.40

Bold accuracy stands for the highest accuracy value

**Table 6:** Average accuracy values from 12:00–24:00 h, using classification models with best hyperparameters.

Classification Model	Accuracy %											
	12:13	13:14	14:15	15:16	16:17	17:18	18:19	19:20	20:21	21:22	22:23	23:24
**SVM**	87.10	86.80	87.50	87.10	87.40	86.90	86.70	87.40	87.10	87.10	87.30	86.60
**Neural Network**	83.50	83.20	83.90	83.40	83.90	83.30	83.20	84.00	84.00	83.90	84.00	83.20
**KNN**	89.20	89.50	89.00	90.00	89.40	89.90	89.60	89.40	90.00	90.00	**90.90**	89.80
**TREE**	88.80	89.50	89.20	88.60	89.50	90.00	89.40	89.40	90.10	90.10	**91.20**	90.00

Bold accuracy stands for the highest accuracy value

**Fig 2:**
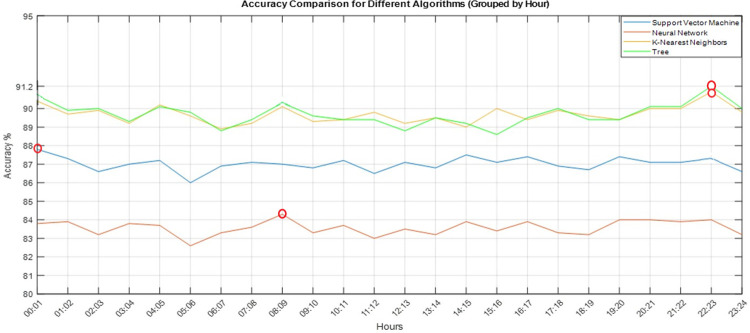
Accuracy per hour utilizing the best hyperparameters combination for the four classification models. The hours of occurrence of the highest values are shown in red circles. Where QTc and QRS are found to be the most important features for predicting HFpEF, HFmEF, and HFrEF categories.

The highest accuracy (accuracy % = 91.20) occurred between 10 pm to 11pm using the Tree classifier, where the maximum depth is 10, and the criterion for splitting is set to Gini. While the KNN classifier led to an accuracy percentage of 90.90 at this time interval, with five neighbours and kd_tree function. The NN classifier’s best performance occurs in the morning interval (8–9 am) with an accuracy percentage of 84.30, where one hidden layer with 100 hidden units, and ReLu activation function with learning rate of 0.01 are set. The early morning time (12–1 am) results in the highest accuracy using the SVM model with a linear function and C value of 10. Furthermore, utilizing the four classification models, Fig 2 shows the accuracy distribution over a 24-hour period. In the latter, the decision tree model surpasses the other models in the three-time intervals; the time interval containing the highest attainable accuracy values is marked with red circle.

**Fig 3:**
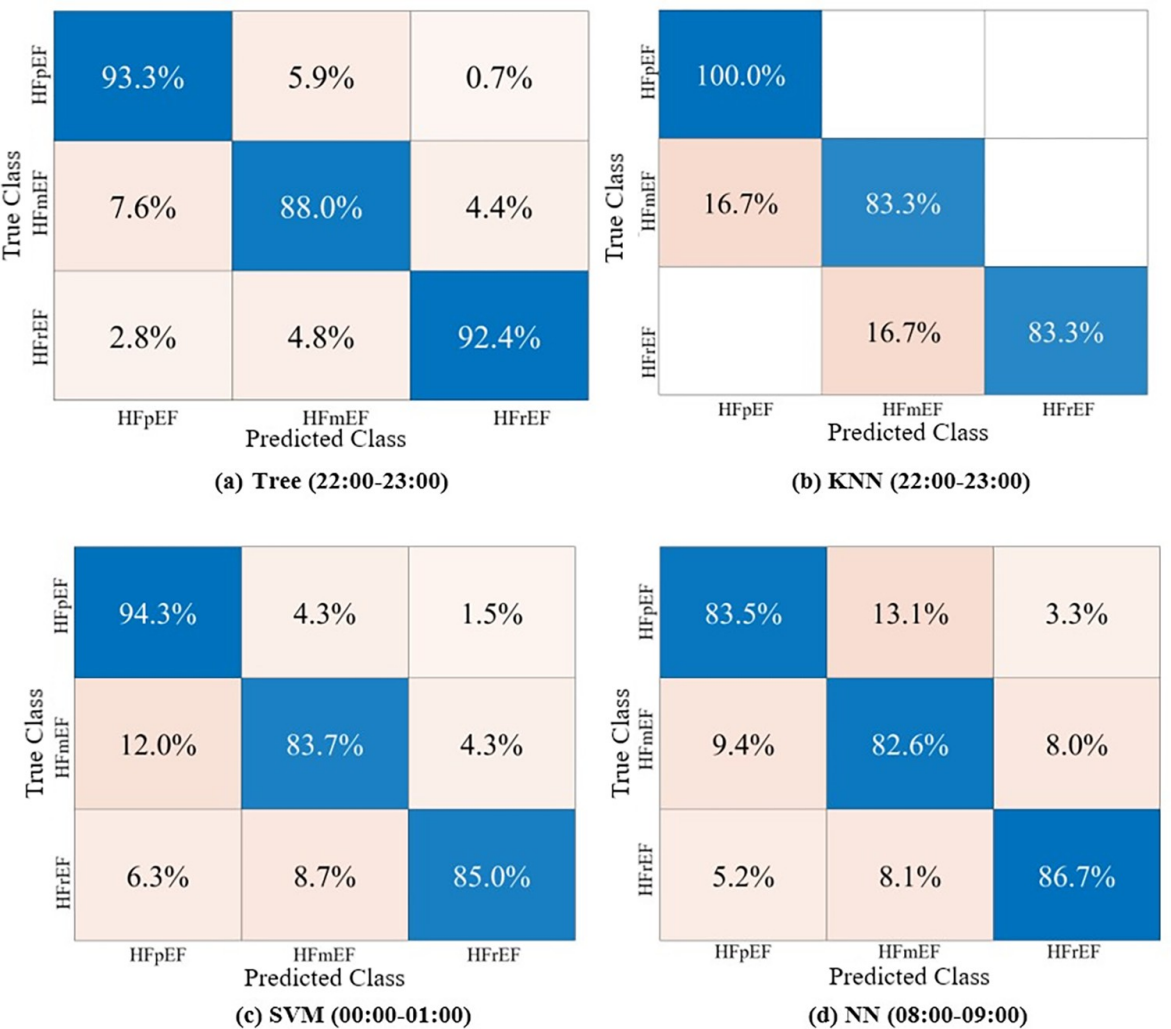
Confusion matrix of the predicted and true classes for hours (22:00–23:00, 00:00–01:00, 08:00–09:00) using the Tree, KNN, SVM, and NN respectively.

**Fig 4:**
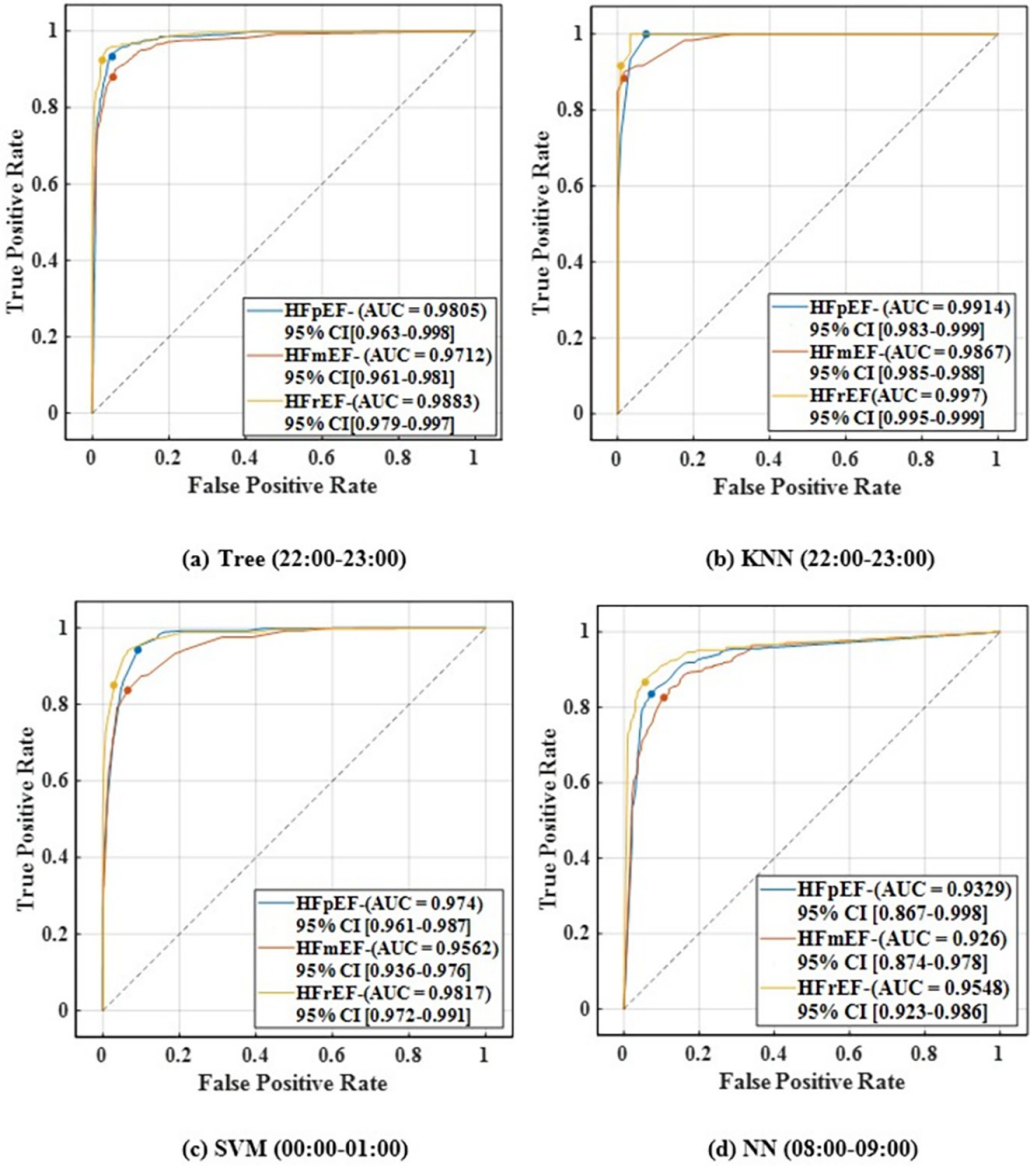
Each LVEF category’s receiver operating characteristics (ROC) curve and the associated area under the ROC curves for hours (22:00–23:00, 00:00–01:00, 08:00–09:00) using the Tree, KNN, SVM, and NN respectively.

**Fig 5:**
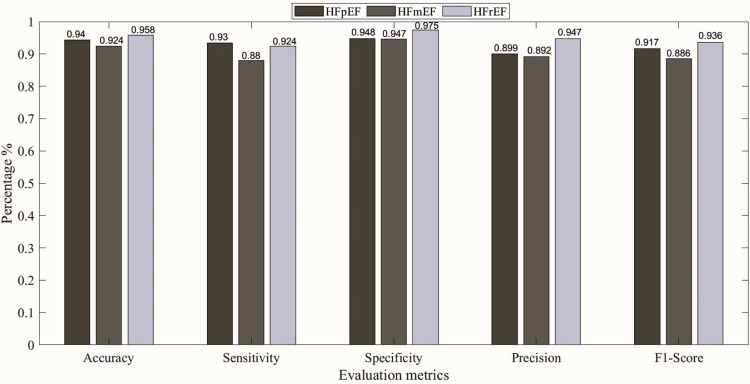
Evaluation metrics for each LVEF category, using Tree at 22:00–23:00 h.

**Fig 6:**
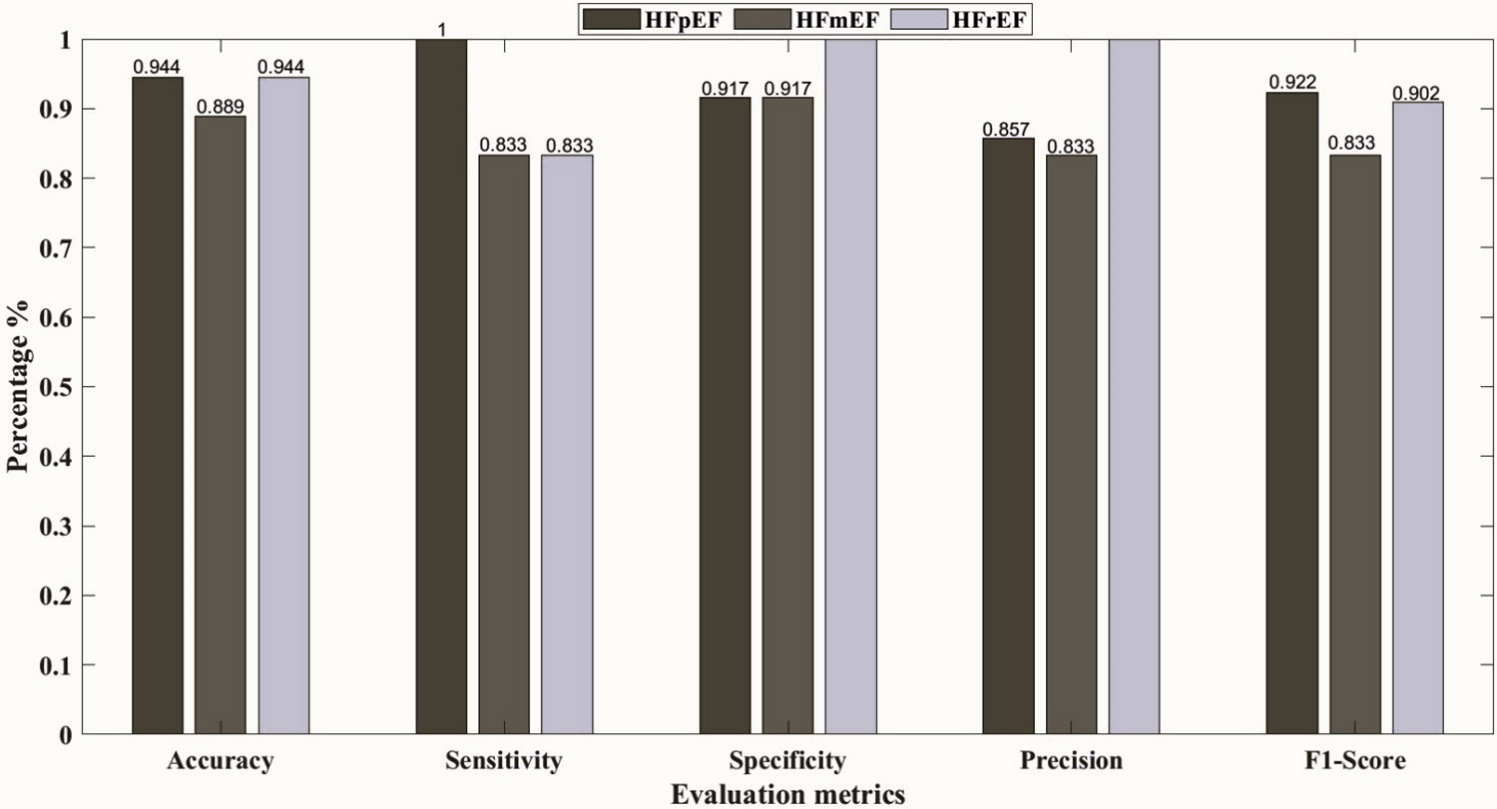
Evaluation metrics for each LVEF category, using KNN at 22:00–23:00 h.

**Fig 7:**
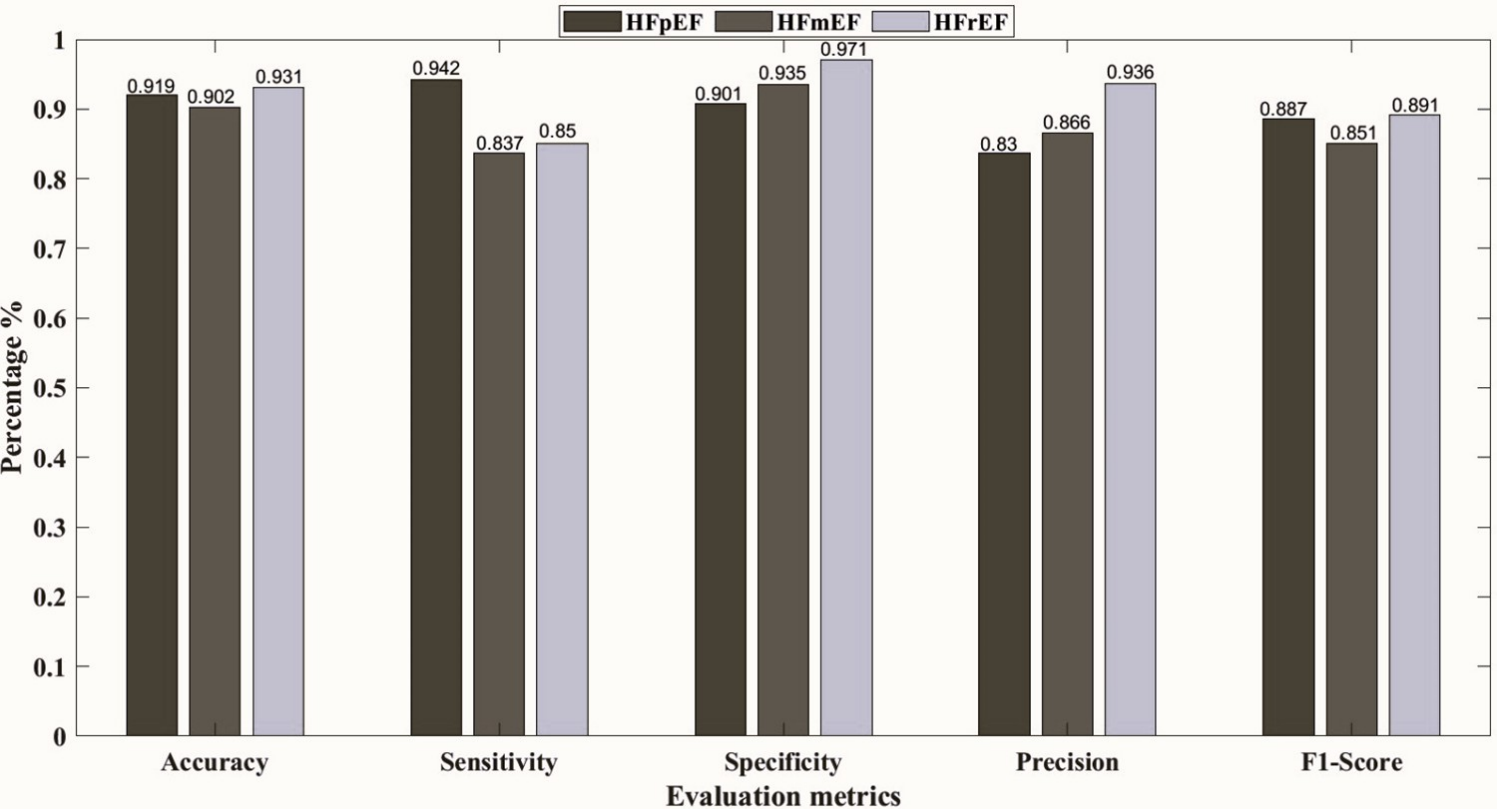
Evaluation metrics for each LVEF category, using SVM at 00:00–01:00 h.

**Fig 8:**
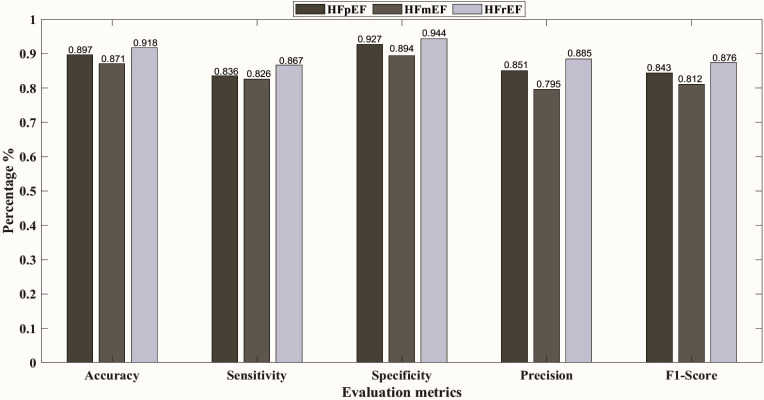
Evaluation metrics for each LVEF category, using NN at 08:00–09:00 h.

Furthermore, Fig 3 shows the confusion matrix representing the classification model’s predictions for each class at 00:00–01:00 h, 08:00–09:00 h, and 22:00–23:00 h with the best results. The decision tree yielded the highest overall accuracy of 91.2% at 10 pm, followed by the KNN model with an overall accuracy of 90.9%. Subsequently, the SVM and NN models achieved overall accuracies of 87.8% at 12 am, and 84.3% at 8 am, respectively. To elaborate more on the prediction models, Figs 4–8 show the ROC curves and the evaluation metrics for each LVEF category, respectively. An average AUROC of 0.98 distributed as 0.9805 for HFpEF, 0.9712 for HFmEF, and 0.9883 for HFrEF resulted in the tree model. Whereas the average AUROC were 0.9917, 0.971 and 0.938 for the KNN, SVM, and NN models, respectively. Moreover, the models demonstrated elevated levels of accuracy, precision, specificity, sensitivity, and F1-score (e.g., Tree model surpassing 88%, other models surpassing 0.79%).

**Fig 9:**
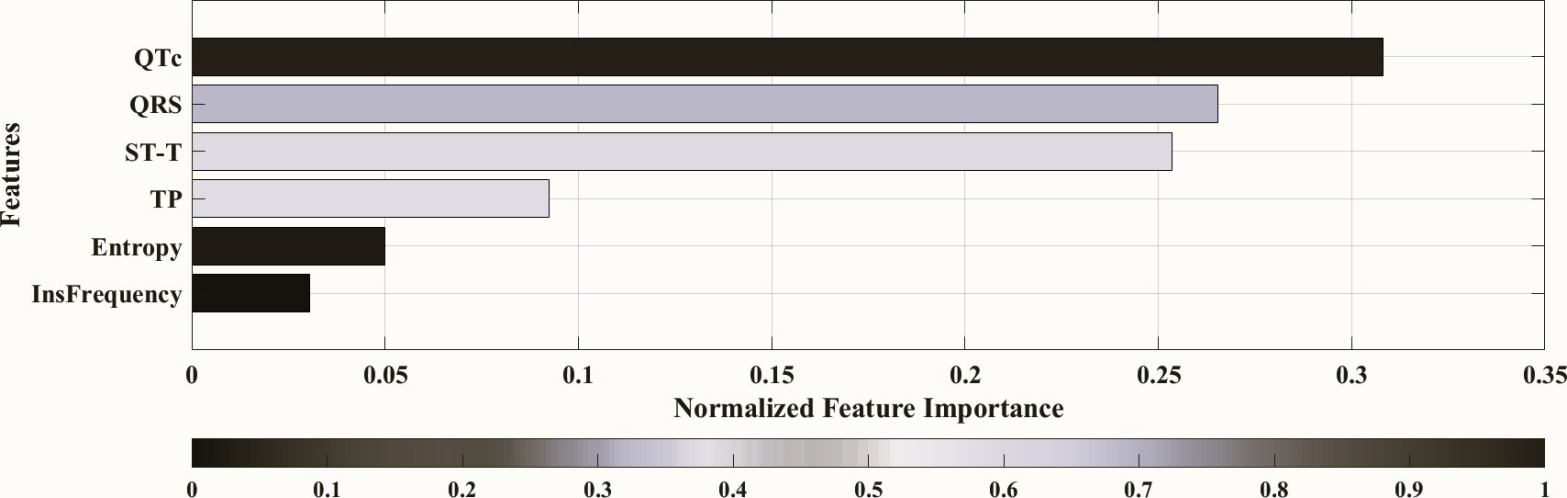
Feature importance over the tree classification model (10:00–11:00pm). This study used importance scores for the ECG features (QTc, QRS, ST-T, TP, Entropy, and Instant Frequency) to classify LVEF levels in the three HF categories. Indicating that QTc is the most important feature for predicting the HFpEF, HFmEF, and HFrEF.

Furthermore, we used the best classification model to assess the feature relevance in this study. The findings are displayed in Fig 9, which emphasizes how important each feature is in relation to how well the model predicts the HF class. Based on their impact on the target variable, the feature importance values have been computed, offering insightful information about the primary factors influencing the LVEF classification. By identifying the most significant features, this study facilitates a more thorough comprehension of the underlying variables affecting the classification model’s result. The thorough assessment of feature relevance facilitates understanding the behaviour of the model and supports well-informed decision-making for our research application.

Moreover, the overall performance of each model is investigated and reported in Table 7. This table provides a comprehensive view of the models performance when considering completely random hour of ECG tracings rather than specific hours.

**Table 7:** Overall performance of each model considering completely random hour of ECG tracings.

Classifier	Overall Accuracy %	F1-score	AUC			Correctly Classified %		
			*HFpEF*	*HFmEF*	*HFrEF*	*HFpEF*	*HFmEF*	*HFrEF*
**SVM**	84.3	0.84	0.93	0.92	0.95	83.5	82.6	86.7
**NN**	81.8	0.80	0.85	0.91	0.86	80.0	83.3	83.3
**KNN**	88.1	0.87	**0.97**	0.95	0.96	94.3	**83.7**	85.0
**TREE**	**90.4**	**0.90**	**0.97**	**0.96**	**0.97**	**94.8**	**83.7**	**91.2**

Bold stands for the highest value

**Fig 10:**
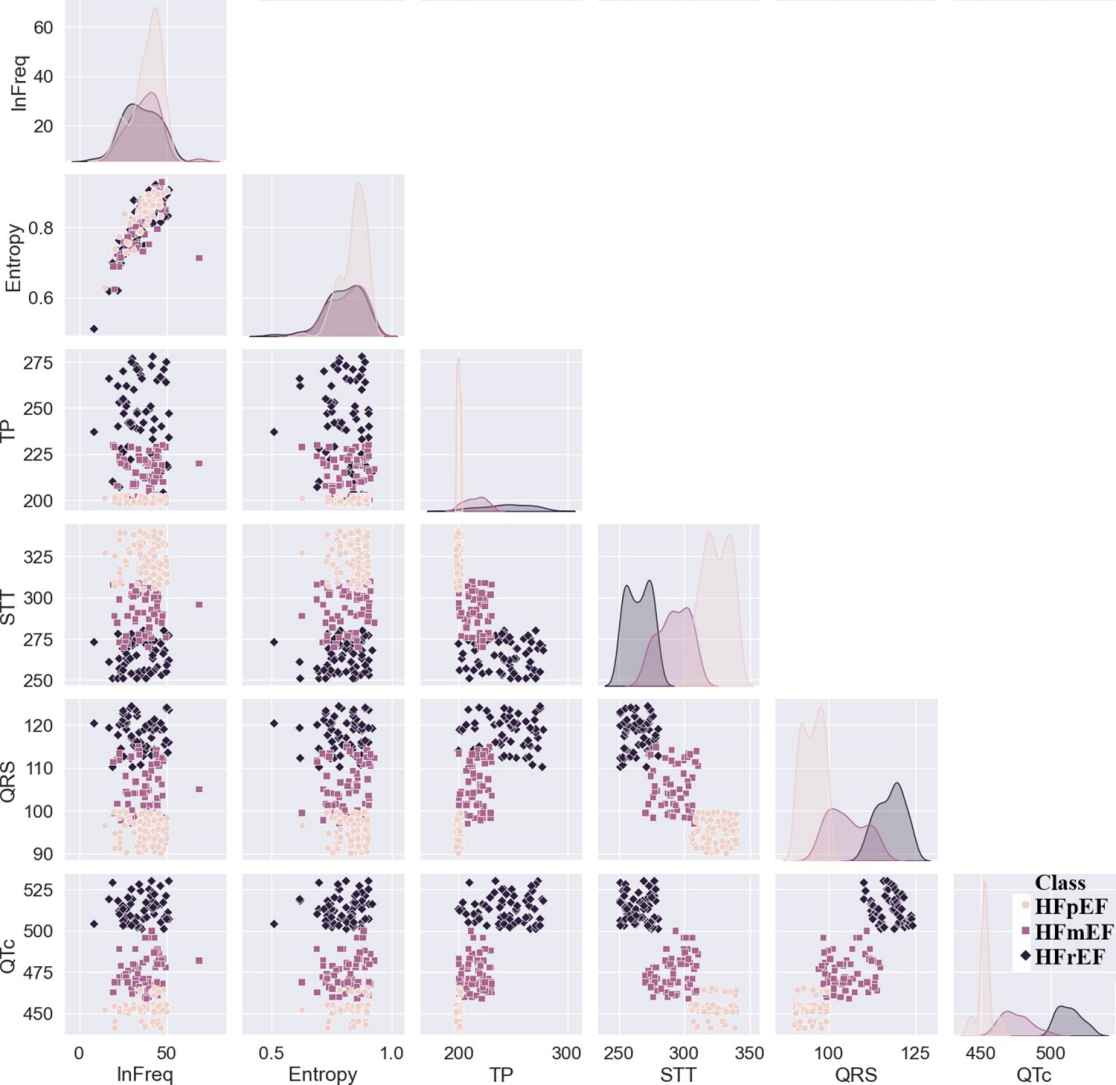
Pair plot matrix of the ECG characteristics and the LVEF categories.

Deeper analysis was done to the relationships and patterns in the employed ECG characteristics, depicted from the pair plot of the six ECG features for an arbitrary selected hour (13:00–14:00) in Fig 10. The pair plot provides a comprehensive view of the interactions between the chosen ECG features, offering insights into potential correlations, trends, and variations. The diagonal plots, represent the univariate distribution of each feature, where QTc and QRS resulted in clearer HFpEF, HFmEF, and HFrEF distribution, respectively. In addition, a noteworthy correlation was identified, particularly between the QTc-QRS and QTc-STT characteristics. This observed relationship signifies a connection between the QTc interval, which reflects the duration of ventricular depolarization and repolarization, and the QRS complex, representing the ventricles’ electrical activation and mechanical contraction. The QTc-QRS correlation suggests that alterations in ventricular conduction, as indicated by QRS duration, may coincide with changes in the duration of ventricular repolarization, represented by the QTc interval.

Similarly, the significant correlation between QTc-STT characteristics highlights how the QTc interval and the STT interval are related. The STT interval is closely related to T-wave morphology, and T-wave abnormalities, such as inversion or flattening, are commonly observed in heart failure patients. Therefore, the QTc-STT correlation implies that variations in the QTc interval may align with changes in the STT interval, providing insights into myocardial ischemia or injury reflected in T-wave patterns.

Overall, these significant correlations contribute to a comprehensive understanding of the interplay among key electrocardiographic parameters, shedding light on potential links between ventricular conduction, repolarization dynamics, and T-wave morphology. This nuanced insight has implications for deciphering the complex physiological mechanisms underlying cardiac performance and dysfunction in the context of HF. This valuable information gained about the interplay between the six ECG features, contributing to a nuanced and data-driven understanding of the underlying physiological and pathological phenomena for predicting the HF patients with distinct LVEF levels.

Each of the 229 models generated, including those using decision tree methods, represents a unique iteration of the model trained on different subsets of the data, as part of the LOOCV process. These models indeed exhibit variability due to the diverse subsets of data used for training, resulting in variations in cutoffs and variable selection at different nodes.

However, it is important to clarify that we did not combine these 229 models into a singular composite decision tree. Instead, our approach involves aggregating the predictions made by each individual model to obtain a final prediction. Remarkably, these aggregated predictions demonstrate high accuracy on average.

**Fig 11:**
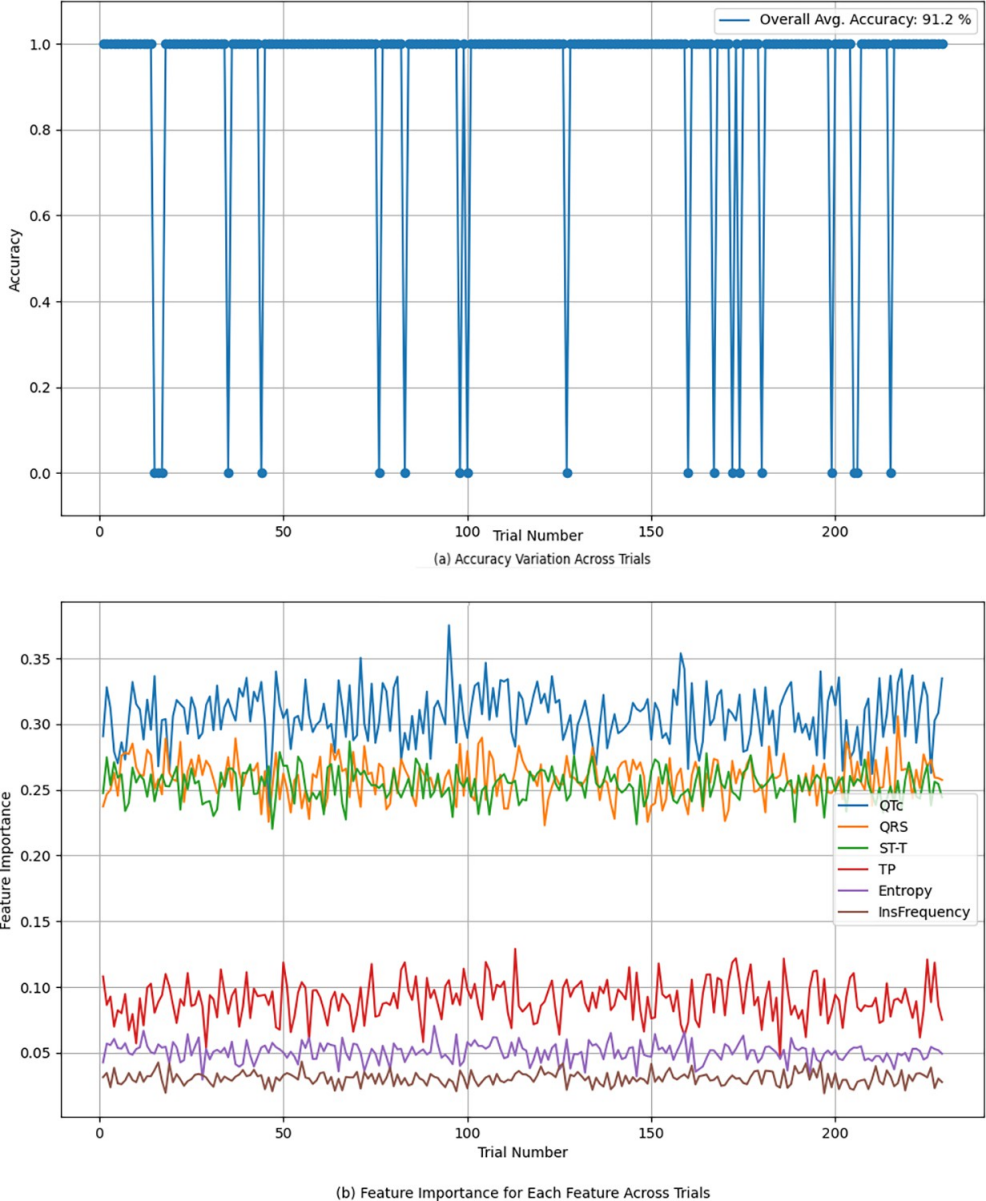
Performance variations across the 229 iterations using the tree method.

To provide a comprehensive understanding of this variability, Fig 11 illustrates the variation in accuracy across the 229 iterations, as well as the variations in feature importance for each feature across the iterations. Notably, the results indicate minimal variability, with standard deviations of [0.021 0.015 0.012 0.016 0.007 0.005], for QTc, QRS, ST-T, TP, Entropy, and InsFrequency, respectively. These small standard deviations highlight the consistency observed across the 229 iterations for each method employed.

## Discussion

This study offers insightful information about the significance of using ECG characteristics to classify heart failure patients according on their LVEF values into HFpEF, HFmEF, and HFrEF groups. As well, the narrower categorization, as outlined in the guidelines from the ASE/EACVI, underscores that even a slight reduction in LVEF can influence heart rhythm and lead to changes in the patient’s overall clinical condition. The study determines the importance of particular ECG patterns in correctly identifying HF categories over the course of a 24-hour examination through a thorough analysis. Utilizing classification analysis, the research aims to enhance precision for optimal predictive performance. Through the identification of ideal hyperparameters and model configurations, the investigation effectively attains elevated accuracy across all four classification scenarios during different phases of the heart’s circadian rhythm. These results showcase the capability of incorporating ECG features into ML algorithms, indicating their potential as valuable tools to aid in screening for heart failure diagnosis and treatment. The temporal characteristics of ventricular systole and diastole are closely reflected in ECG parameters like QRS duration, T-wave abnormalities, and ST segment alterations. Similarly, the QTc interval, representing ventricular repolarization, intricately mirrors the duration needed for myocardial cells to return to their resting state after depolarization. The prediction of LVEF category, which indicates the effectiveness of ventricular contraction, is inherently connected to the temporal aspects of cardiac repolarization as indicated by the QTc interval. Likewise, the QRS duration signifies the transmission of electrical impulses during ventricular depolarization, intricately linked to mechanical processes. Alterations in QRS duration may indicate changes in ventricular conduction, impacting the synchronization of contraction and subsequently influencing LVEF. By leveraging the electrophysiological details embedded in ECG features, the models take advantage of the profound relationship between electrical signaling and mechanical performance. This collaboration provides a more comprehensive insight into cardiac function, enabling the models to adeptly categorize HF patients based on their LVEF levels into HFpEF, HFmEF, and HFrEF groups with enhanced accuracy. This precision stems from the intricate interplay between electrophysiological dynamics and mechanical behavior.

In contrast to prior studies (Table 8), Bi-Directional Long Short-Term Memory (BiLSTM) classifier was employed to categorize LVEF groups of CAD patients applying swarm decomposition components on their heart rate variability. The corresponding oscillatory four bands components (HRV-OCs) were derived from 24-hour ECG recordings of 271 American cohorts [48]. The patients were categorized based on their LVEF as border line, normal, or at risk. The BiLSTM model exhibited varying performance throughout the 24 hours. The most accurate classification, with an average accuracy of 75.6%, was achieved during the time interval between 3 am and 4 am. The very-low frequency components of the HRV were employed for this interval, and achieved sensitivity levels of 76.7% in predicting the border line group. In a different investigation [49], three distinct models (e.g., Generalized Linear Model (GLM), SVM with RBF kernel, and CNN), were utilized to discern between HF patients categorized into three specific groups. The study utilized clinical profiles of 303 CAD patients, sourced from Greek and American patient databases. The objective was to compare the efficacy of the three models in classifying HF patients into HFpEF, HFmEF, and HFrEF in accordance with the ASE/EACVI guidelines. Following thorough evaluation, it was noted that the CNN model outperformed the other methods, achieving an overall accuracy of 90.1%, whereas the resultant overall accuracy was 84.14% and 88.45% using GLM and SVM, respectively.

**Table 8:** Summary of the studies for LVEF classification in preserved, midrange, and reduced HF patients. Bold classifier indicates the best performance.

Author (Year)	ECG Signal Duration	Data	Method/Features	Classifier	Overall Acc. %
**[48] 2021**	1hour (24h study)	271 (American Cohort)	Swarm Decomposition oscillatory components of HRV	LSTM	75.6
[49] 2021	NA	303 (American, Greek Cohorts)	Clinical Profiles	CNN SVM Generalized linear Model	90.10 88.45 84.14
**Current study**	**1hour (24h study)**	**229 (American, Greek Cohorts)**	**ECG Features**	**TREE** KNN SVM NN	**91.2** 90.9 87.8 84.3

The present study surpasses the performance of previous works [48, 49] in the literature for classifying HF patients into HFpEF, HFmEF, and HFrEF from ECG features only. The results of this investigation reveal that the optimal performance is achieved during the time interval between 10 pm and 11 pm, using a tree-based model. This model exhibits an impressive overall accuracy of 90.2%, and higher precision levels in HFrEF prediction with 89.2%, surpassing the KNN, SVM, and NN performance metrics.

### Clinical relevance and ECG features significance

ECG characteristics derived from continuous ambulatory Holter monitoring have proven to be valuable indicators for both total mortality and the advancement of heart failure. This ongoing monitoring method provides distinctive perspectives into a patient’s cardiac well-being, enabling a thorough assessment of heart rhythm and electrical activity over an extended duration. Even when considering other recognized risk factors (e.g., age, gender, and comorbidities), the data obtained from Holter monitoring contributes supplementary and significant prognostic details. Nevertheless, LVEF categorization in HF patients on an hourly basis, using ECG features, can provide valuable observations into the temporal trends and variations in cardiac function. The analysis of LVEF levels hour-by-hour aimed to explore potential variations in model performance over time rather than suggesting a definitive prediction at an hourly resolution. The variability observed in accuracy reflects the dynamic nature of the data, and our goal was to assess how well the models adapt to different segments of the ECG tracings. This insight into temporal variations becomes crucial for adapting treatment plans or interventions according to the patient’s circadian rhythm and physiological shifts. This study showcases exceptional performance in distinguishing LVEF levels, particularly during the evening (22–23) and early morning hours (12 am-1 and 8–9). Moreover, these specific hourly intervals correspond to previously documented high-risk periods associated with elevated mortality and incidences of myocardial infarctions in the early morning and evening hours [50–52].

Additionally, with the application of the most efficient classification model, we conducted an assessment of feature significance in this investigation. The findings showed that, in decreasing order of significance, QTc, QRS, ST, and TP are the most important characteristics affecting the classification of LVEF in patients with heart failure. These distinct ECG parameters exerted a substantial impact on the target variable, offering valuable insights into characterizing cardiac function and underscoring their potential as pivotal clinical indicators for the evaluation of HF patients. The significance of QTc as the foremost feature can be ascribed to several critical factors [53–56]. The QTc interval indicates the timeframe of ventricular repolarization and depolarization, encompassing the electrical conduction duration within the heart. In individuals with heart failure (HF), disruptions in electrical conduction often occur as a result of ventricular remodeling and shifts in ion channel kinetics. Extended QTc intervals may indicate delayed repolarization, a factor associated with compromised ventricular health involving changes in ventricular size, shape, and function, leading to a reduction in LVEF. Patients with HF face an elevated risk of arrhythmias, and the prolongation of QTc is a recognized risk factor for severe ventricular arrhythmias. Irregular QTc intervals indicate the presence of an arrhythmogenic substrate and can impact LVEF by potentially triggering life-threatening arrhythmias that can disrupt cardiac function. Moreover, the QTc interval is influenced by autonomic nervous system activity, particularly the inputs from the parasympathetic and sympathetic systems to the heart. HF patients commonly experience altered autonomic regulation, and QTc prolongation may be associated with autonomic dysfunction, thereby influencing cardiac function and LVEF. Certain medications frequently prescribed for HF management have the potential to influence the QTc interval. The QTc-prolonging impact of specific medications may underscore the significance of this feature in the model, as the consideration of medication usage is a crucial factor in distinguishing LVEF.

Many studies have demonstrated that QRS duration serves as a prognostic indicator in HF patients [57–59]. The QRS complex represents the electrical activation and subsequent mechanical contraction of the ventricles. In HF patients, mechanical desynchrony is a prevalent occurrence, characterized by uncoordinated contractions in different ventricular regions. Prolonged QRS duration may signal the presence of bundle branch blocks (BBBs), indicating conduction abnormalities that can impact ventricular function and contribute to a reduction in LVEF [60]. Additionally, for individuals undergoing cardiac resynchronization therapy (CRT), QRS duration stands as a crucial parameter for assessing eligibility for CRT implantation. CRT effectively enhances mechanical synchrony in HF patients with electrical desynchrony and prolonged QRS duration. By incorporating QRS duration into HFpEF, HFmEF, and HFrEF diagnosis, clinicians can obtain insights into potential CRT suitability and tailor treatment strategies for improved patient outcomes.

Furthermore, the STT interval exhibits a close relationship with T-wave morphology. T-wave abnormalities, including inversion or flattening, are frequently observed in individuals with heart failure [61–64]. These alterations in T-wave patterns can provide valuable insights into myocardial ischemia or injury and contribute to the accuracy of differentiating LVEF levels. Irregularities in the TP interval may indicate anomalies in repolarization, which can be linked to compromised cardiac function in HF patients. Such deviations have been correlated with an elevated risk of arrhythmias, such as ventricular fibrillation and ventricular tachycardia. Numerous studies have established a connection between abnormal TP intervals and a decline in LVEF among HF patients. Prolonged TP intervals have been associated with impaired ventricular function, while shortened TP intervals may signify myocardial remodeling and potential deterioration of cardiac performance.

The emphasis on these particular features, guided by their influence on the classification of HFpEF, HFmEF, and HFrEF, underscores the model’s proficiency in identifying significant patterns and connections within diverse ECG parameters. This comprehension is crucial for advancing our insight into the fundamental physiological mechanisms governing both cardiac well-being and dysfunction.

## Limitations and future work

While ML-based models have demonstrated efficacy in predicting LVEF, our study acknowledges several limitations inherent in these models. It’s important to acknowledge the limitations of any single metric in representing the entirety of heart failure classification. The complexity of heart failure classification requires a comprehensive consideration of multiple parameters and diagnostic measures for a more accurate and reliable assessment. Our study emphasizes the need for a holistic approach to ensure robust and clinically meaningful categorization of heart failure patients. The datasets do not include recorded or available information regarding the effects of specific drugs such as amiodarone, ranolazine, etc., on the QT interval. As a result, we were unable to control for the potential impact of these medications on QT intervals in our analysis. Initially, our focus centered on utilizing six features extracted from the circadian ECG for heart failure (HF) categorization. However, there is a necessity for additional exploration and scrutiny of additional features, including but not limited to QRS area, amplitude of peak changes, and their correlation with EDR (ECG-derived respiration). This broader investigation is crucial for obtaining more comprehensive insights into the influence of these features on LVEF predictions. Moreover, although the present investigation employed a dataset encompassing patients from both American and Greek populations, it is imperative to subject the trained models to additional testing on more diverse patient groups to ensure a wider applicability and generalization of their performance. The current patient cohort exhibits a notably higher representation of male participants compared to females. Subsequent studies could undertake further examinations of LVEF categorization on a more uniformly dispersed data, balancing the representation of male and female subjects. In future investigations, the classification models formulated in this study might hold promise for adaptation or extension to predict diverse parameters associated with heart health, such as myocardial ischemia. However, their suitability for underlying conditions that may contribute to heart failure necessitates further research and validation.

While our study delves into patterns within a single 24-hour Holter trace per patient, we recognize the inherent variability in circadian rhythms among individuals. Ideally, multiple traces over different days would offer a more comprehensive view. However, practical constraints limit our focus to single-trace data. Additionally, the datasets did not include information about the sleep apnea status of the patients, this prevents us from making specific observations or drawing conclusions about the influence of obstructive sleep apnea on the studied parameters.

## Conclusion

This investigation underscores the promise of utilizing ECG features derived from 24-hour recordings as an efficient complementary tool to the established echocardiography gold standard for categorizing individuals with heart failure in one of the three LVEF groups. From a ML standpoint, the devised approach provides valuable insights into identifying ECG patterns conducive to precisely categorizing LVEF within distinct HF categories, namely preserved, midrange, and reduced. Moreover, the envisioned research seeks to broaden the scope of LVEF assessment to communities facing limitations in accessing the necessary instruments, whether due to economic constraints or a shortage of clinical expertise. The prospective findings of this investigation could assist in the development of a model that can predict the HF phenotype or monitoring its evolution during therapy. Consequently, this proposed approach could function as a versatile instrument for exploring the pathophysiology of the disease and objectively assessing therapeutic strategies in potential HF patients. Notably, the suggested ML model is characterized by simplicity, efficiency, and cost-effectiveness, allowing for continuous cardiac analysis and serving as a viable adjunct to more intricate gold standard techniques. As research progresses, the utilization of more extensive datasets and the incorporation of deep learning techniques will facilitate a comparison of the proposed model’s performance against other methodologies. In essence, this study contributes to the advancement of ECG-based HF classification and its potential applicability, particularly in resource-constrained settings.
